# The effect of adjunctive chlorhexidine mouthrinse on GCF MMP-8 and TIMP-1 levels in gingivitis: a randomized placebo-controlled study

**DOI:** 10.1186/1472-6831-14-55

**Published:** 2014-05-20

**Authors:** Oya Türkoğlu, Sema Becerik, Taina Tervahartiala, Timo Sorsa, Gül Atilla, Gülnur Emingil

**Affiliations:** 1 Department of Periodontology, Ege University, School of Dentistry, Izmir, Turkey; 2 Department of Oral and Maxillofacial Diseases, Helsinki University Central Hospital, Institute of Dentistry, University of Helsinki, Helsinki, Finland; 3 Division of Periodontology, Department of Dental Medicine, Karolinska Institutue, Stockholm, Sweden

**Keywords:** Chlorhexidine, Gingivitis, Gingival crevicular fluid, MMP-8, TIMP-1

## Abstract

**Background:**

The aim of the present study was to evaluate the effect of adjunctive chlorhexidine (CHX) mouthrinse on gingival crevicular fluid (GCF) MMP-8 and TIMP-1 levels in plaque-associated gingivitis.

**Methods:**

A total of 50 gingivitis patients were included in the present study. In addition to daily plaque control, CHX group rinsed with CHX, while placebo group rinsed with placebo mouthrinse for 4 weeks. GCF samples were collected, and clinical parameters including plaque index, papillary bleeding index, calculus index and pocket depth were recorded at baseline and 4 weeks. GCF MMP-8 and TIMP-1 levels were determined by immunofluorometric assay (IFMA) and enzyme-linked immunosorbent assay (ELISA), respectively.

**Results:**

In both groups, GCF MMP-8 levels of anterior and posterior sites at four weeks were not different from baseline (p > 0.05). There were no significant differences in GCF MMP-8 levels between the study groups at four weeks (p > 0.05). GCF TIMP-1 levels of anterior and posterior sites at four weeks were higher compared to baseline in both groups (p < 0.05). There was no significant difference in GCF TIMP level between the study groups at four weeks (p > 0.05).

**Conclusions:**

CHX usage had no significant effects on the GCF MMP-8 and TIMP-1 levels in plaque-associate gingivitis. However, daily plaque control resulted in the increase of GCF TIMP-1 levels regardless of CHX usage.

## Background

Dental plaque is the major etiological factor associated with the development of gingivitis [1]. Mostly, efficient mechanical plaque control would be enough for the resolution of the gingival inflammation. Antimicrobial mouthrinses as adjuncts to daily plaque control is more beneficial than only brushing when individuals are unable to consistently maintain adequate levels of plaque control using mechanical methods alone [2,3]. Chlorhexidine (CHX) mouthrinse as antimicrobial agent is considered as the gold standard in preventing the dental plaque formation and gingival inflammation due to its both antiplaque and antigingivitis effects [4-8].

Microorganisms of dental plaque stimulate host cells to express their matrix metalloproteinases (MMPs), which is one of the mechanisms leading to periodontal tissue destruction [9]. MMP-8 is expressed by many types of cells [9,10] and known to be the main host cell-derived collagenase that contributes significantly to tissue destruction and remodeling events in inflammatory periodontal diseases [11-14]. Significant decreases in gingival crevicular fluid (GCF) MMP-8 levels following periodontal treatment including scaling and root planning have been reported [15]. However, there are limited data related to the MMP-8 levels in gingivitis patients [16-18]. Emingil et al. [17] demonstrated GCF MMP-8 total amount to be significantly higher in gingivitis patients compared to healthy controls. Atilla et al. [16] found no significant difference in GCF MMP-8 levels between gingivitis and control subjects. Recently, the persistence of MMP-8 at physiologic levels after treatment has been suggested to be involved in the down-regulation of the inflammatory process and the onset of the reparative phase [19].

MMP activity is regulated by changes in the balance between the expression and synthesis of MMPs and tissue inhibitors of matrix metalloproteinases (TIMPs) [9]. TIMP-1 was demonstrated to be the major inhibitor of MMPs in gingival tissues of patients with periodontal disease [20-22]. The balance between MMPs and TIMPs plays an essential role in maintaining the healthy condition of periodontal tissues, and disturbed balance may cause collagen breakdown and periodontal tissue destruction. It has been demonstrated that periodontal treatment increased TIMP-1 expression and decreased the ratios of MMPs/TIMP-1 in chronic periodontitis [23,24]. However, previous studies have demonstrated controversial results about TIMP-1 levels in periodontal disease. Both increased and decreased TIMP-1 levels were reported in GCF and gingival tissues of patients with periodontitis [20-22,25-30].

We have previously showed CHX mouthrinse had limited beneficial effects on clinical periodontal parameters but was not effective on GCF cytokine levels in gingivitis [31]. It has been stated that CHX mouthrinses is very efficient agent in reducing and/or preventing dental plaque [32-34] and had an additional effect on the degree of inflammation in oral cavity [3,6,32,34,35]. CHX can inhibit MMPs and prevent oxidative activation of MMPs and oxidative inactivation of α1-antitrypsin [36-38]. TIMP-1 can also be inactivated by reactive oxygen species [9]. Therefore, we hypothesized that the reduction of dental plaque by CHX mouthrinse might affect the GCF MMP-8 and TIMP-1 levels, which is known to act as crucial mediators in inflammatory process of periodontal diseases. To the best of our knowledge, there is no study investigating the efficacy of CHX mouthrinse in addition to daily plaque control on GCF MMP-8 and TIMP-1 levels in the presence of plaque-associated gingivitis. Therefore, the present study aimed to determine the effect of adjunctive chlorhexidine mouthrinse on GCF MMP-8 and TIMP-1 levels in plaque-associated gingivitis.

## Methods

### Study population

A total of 50 gingivitis patients who were diagnosed according to AAP consensus report [39] were included in the present study. All consecutive subjects were recruited from the Ege University, School of Dentistry, Department of Periodontology over a period of 1 year between 2006 and 2007. The study protocol was approved by the ethics committee of the Ege University School of Medicine. Prior to participation, the purpose and procedures were fully explained to all patients and all participants gave written informed consent in accordance with Helsinki declaration. The study was designed, conducted, analysed and reported according to guidelines for Good Clinical Practice.

Medical and dental histories were taken at pre-screening visit. Inclusion criteria were: 18–45 years of age, male or female patients with gingivitis associated with dental plaque, clinical attachment level <3 mm, a minimum of 20 teeth (teeth that have gross caries, were fully crowned or extensively restored, orthodontic banded, abutments, or third molars were not included in the tooth count). Exclusion criteria were as follows: use of tobacco products, history or current manifestation of systemic disease that could impair immune response such as diabetes mellitus, immunological disorders, hepatitis and HIV infections, use of antibiotic or anti-inflammatory or immunosuppressive drugs during 3 months period prior to the start of the trial, periodontal therapy during the last 3 months, pregnancy or lactation and oral contraceptives usage.

### Study design

This clinical trial was a randomized, double-blind, placebo-controlled, parallel-group study of four week duration. The clinical trial was organized into 4 stages including pre-screening, screening, baseline, and evaluation. Each volunteer was subjected to a pre-screening examination to assess the overall status of his/her dentition. During the pre-screening visit, clinical and radiographic evaluations were assessed for suitability to be included in the study. A single examiner determined assessment of patient eligibility for the study and enrolment of patients into trial. Patients eligible for the study returned to our clinic at screening visit in order to record clinical parameters 2 days after pre-screening. A single experienced examiner assessed the periodontal status of each patient. The same examiner recorded all clinical measurements over the course of the study that was unaware of the type of mouthrinse (CHX or placebo) provided to the patient. After being selected for the study, the subjects who were unaware of the type of mountrinses were randomized either to CHX or to placebo groups. The placebo mouthrinse was composed of CHX mouthrinse ingredients except that it lacked the active ingredients (chlorhexidine digluconate). Both CHX and placebo bottles were similar in appearance.

At the baseline stage of the study the GCF samples were collected. Following completion of the GCF sampling, patients were randomly assigned to CHX or placebo groups by taking into account the gender, age and the extent of the gingivitis by an independent periodontist who kept the allocation information confident until the data collection and biochemical analysis were completed. The extent of gingivitis was assessed according to number of the bleeding papilla and the severity of the bleeding. Only the patients who have at least 30% of the papillae with papillary bleeding index (PBI) 2 or 3 were included in the present study. The subjects were given a fluoride containing dentifrice (Ipana®, P&G GmbH, Germany) and toothbrush (Oral B indicator®, P&G, Ireland) for use during the study period. All of the patients received tooth brushing instructions of Modified Bass technique and brushed their teeth twice a day with toothbrush and toothpaste given for four weeks. Thirty minutes after toothbrushing, CHX group rinsed with 10 ml of 0.2% CHX mouthrinse twice a day (once in the morning and once in the evening before sleeping) for 1 min. Thirty minutes after toothbrushing, placebo group rinsed with 10 ml of placebo mouthrinse twice a day (once in the morning and once in the evening before sleeping) for 1 min. They also had to avoid rinsing afterwards with water, as well as eating or drinking within 30 min. following the use of the mouthrinse. All patients came to control visits once per week and returned their mouthrinse bottles to the study supervisor. The side effects of the mouthrinses were reviewed every week during the study period. If any painful side effect such as mucosal ulcerations was observed, utilization of mouthrinses was broken and the patient was excluded from the study. At the end of the 4 weeks period, GCF samples were taken and clinical recordings were repeated.

### Clinical assessment

Clinical periodontal parameters including plaque index (PI) [40], PBI [41], which were primary outcomes of the present study, calculus index (CI) [42] and probing pocket depth (PPD) were recorded at six sites of each tooth. All measurements were performed by a single blinded and calibrated examiner using a manual Williams periodontal probe (Hu-Friedy, Chicago, IL). The intra-examiner reliability was high as was revealed by intraclass correlation coefficient 0.87 for PPD measurements and 0.85 for PI measurements.

### GCF sampling

GCF samples were taken from mesiobuccal aspects of a single rooted tooth (except premolars) and a multi-rooted tooth in each subject. Prior to GCF sampling, the supragingival plaque was removed from the interproximal surfaces with a sterile curette; these surfaces were dried gently by an air syringe and were isolated by cotton rolls. GCF was sampled with filter paper (Periopaper, ProFlow, Inc., Amityville, NY). Paper strips were carefully inserted 1 mm into the crevice and left there for 30 seconds [43]. Care was taken to avoid mechanical injury. Strips contaminated with blood were discarded [44]. The absorbed GCF volume of each strip was determined by an electronic device (Periotron 8000, ProFlow, Inc., Amityville, NY) and placed into a sterile polypropylene tube and kept at -80C until being analysed. The readings from the Periotron 8000 were converted to an actual volume (μl) by reference to the standard curve.

### Immunofluorometric assay (IFMA) for MMP-8 and enzyme linked immunosorbent assay (ELISA) for TIMP-1

The secondary outcomes of the present study were the changes of the GCF MMP-8 and TIMP-1 levels. The amounts of GCF MMP-8 were assayed by time-resolved IFMA (Medix Biochemica, Kauniainen, Finland) [45]. IFMA utilizes two monoclonal anti-MMP-8 antibodies and exerts higher accuracy. The antibody identifies the neutrophil- and fibroblast-type MMP-8 isoforms and particularly their active forms in IFMA technique. GCF TIMP-1 was assayed by commercially available ELISA kit (Amersham Biotrak, GE Healthcare, Buckinghamshire, UK). Prior to the quantitation of MMP-8 and TIMP-1, GCF samples were eluted from each strip into 75 μl of 50 mM Tris–HCl-buffer, pH 7.8, containing 0.2 M NaCl and 1 mM CaCl_2_ for 2 h at 22°C on a shaker. The minimum detection limits of MMP-8 and TIMP-1 were 0.08 ng/ml and 1.25 ng/ml, respectively. All samples were assayed in duplicate. The amounts of MMP-8 and TIMP-1 in each sample were calculated based on the dilutions.

### Statistical analysis

The power calculation analysis revealed that the required sample size was minimum 15 subjects for each study group. Sample size of the present study was calculated to detect a 0.5 difference in PI and PBI scores at the 0.05 probability level with a power of 80%. Decision about whether to use parametric or non-parametric tests were made based on the results of Kolmogorow-Smirnow test for normal distribution.

Intragroup comparisons of the GCF MMP-8 and TIMP-1 levels and the clinical parameters of the study sites between baseline and four weeks were tested by Wilcoxon signed rank test to analyze the significance of changes over time. The Mann–Whitney test was used to determine significant differences in GCF MMP-8 and TIMP-1 levels and the clinical parameters of the study sites between the CHX and placebo groups.

## Results

### Patient disposition and demographics

At the pre-screening stage of the present study, two hundred and fifteen patients were examined. Ninety-two patients did not fulfill the inclusion criteria and 36 patients did not accept to attend the study. Finally, a total of 87 gingivitis patients who fulfilled all inclusion criteria were included into the study. Random assignment resulted in 45 patients in the CHX group and 42 patients in the placebo group.

**Figure 1:**
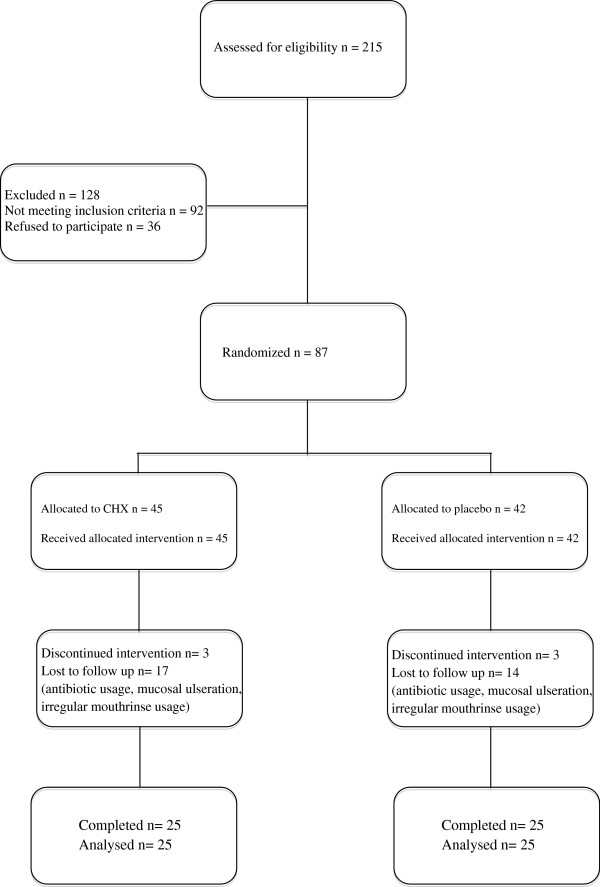
Flow chart of participation in the study.

Thirty-seven patients were excluded from the study during clinical trial because of the following reasons: seven patients in CHX group and nine patients in placebo group who did not use the mouthrinse regularly; three patients in CHX group and three patients in placebo group who discontinued the study; seven patients in CHX group and four patients in placebo group who received antibiotics during the experimental period; three from CHX and one from placebo group who had mucosal ulcerations. Finally, a total of 50 patients, 25 from the CHX group and 25 from the placebo group, completed the 4 weeks clinical trial (Figure 1). Intent to treat analysis was not performed as data from non-compliant patients had only baseline data. Statistical analysis was performed on data from 50 patients who completed the trial. There were no differences between CHX and placebo groups in the age, gender, and the distribution of the extent of gingivitis at the baseline (Table 1).

**Table 1:** Demographic data of the study groups

**Parameters**	**CHX group**	**Placebo group**
**N**	25	25
**Age (years)**		
**(mean ± SD)**	27.56 ± 8.3	25.44 ± 5.6
**Gender**		
**Female/male**	12/13	13/12
**Extent of gingivitis**		
PBI ≥ 2 at 8–13 sites	11	12
PBI ≥ 2 at more than 13 sites	14	13

CHX: Chlorhexidine.

PBI: Papillary bleeding index.

SD: standard deviation.

### Clinical periodontal parameters of whole mouth

The details of clinical findings of whole mouth were reported previously [31]. Briefly, both treatment strategies resulted in similar statistically and clinically significant improvements on the basis of whole mouth PBI, PI, and PPD values at 4 weeks when compared with baseline levels. However, CHX group showed lower PI values than the placebo group at 4 weeks (p < 0.05) and the reductions in the PI from baseline were significantly greater in the CHX group (p < 0.05).

### Clinical periodontal parameters of sampling sites

There were no significant differences in clinical periodontal parameters and GCF values of both anterior and posterior sampling sites between CHX and placebo groups at baseline (p > 0.05). No statistically significant difference was detected in periodontal parameters including PPD, PBI, PI, CI and GCF values of anterior sampling sites at 4 weeks compared to baseline in both groups (p > 0.05) (Table 2). There was no statistically significant difference in PPD, PBI and CI and GCF values of posterior sampling sites of patients in both CHX and placebo groups at 4 weeks compared to baseline (p > 0.05) (Table 3). Posterior sampling sites of CHX group had significantly lower PI values compared to those sites of placebo group at four weeks (p *<* 0.05) (Table 3).

**Table 2:** Clinical periodontal parameters of anterior sampling sites in CHX and placebo groups

**Parameters**	**CHX group**		**Placebo group**	
	**Baseline**	**4th week**	**Baseline**	**4th week**
**PPD (mm)**	3 (1–4)	2 (1–3)	3 (2–5)	3 (1–4)
**PBI**	2 (2–3)	1 (0–3)	2 (2–3)	1 (0–3)
**PI**	4 (1–5)	2 (0–4)	4 (2–5)	3 (1–5)
**CI**	1 (0–2)	2 (0–2)	0 (0–2)	2 (0–2)
**GCF (μl)**	0.19 ± 0.08	0.18 ± 0.2	0.20 ± 0.12	0.17 ± 0.1

Data are given as mean ± SD or median (min-max).

CHX: Chlorhexidine.

PPD: Probing Pocket Depth, PBI: Papillary Bleeding Index, PI: Plaque Index, CI: Calculus Index, GCF: Gingical Crevicular Fluid.

**Table 3:** Clinical periodontal parameters of posterior sampling sites in CHX and placebo groups

**Parameters**	**CHX group**		**Placebo group**	
	**Baseline**	**4th week**	**Baseline**	**4th week**
**PPD (mm)**	3 (2–5)	3 (1–5)	3 (2–5)	3 (2–5)
**PBI**	2 (2–3)	1 (0–3)	2 (2–3)	1 (0–3)
**PI**	4 (2–5)	2 (0–4)*	4 (2–5)	3 (1–4)
**CI**	2 (0–2)	2 (0–2)	2 (0–2)	2 (0–2)
**GCF (μl)**	0.27 ± 0.15	0.17 ± 0.15	0.32 ± 0.22	0.20 ± 0.13

Data are given as mean ± SD or median (min-max).

*Significant difference from placebo at 4 weeks (p < 0.05).

CHX: Chlorhexidine.

PPD: Probing Pocket Depth, PBI: Papillary Bleeding Index, PI: Plaque Index, CI: Calculus Index, GCF: Gingical Crevicular Fluid.

### Laboratory findings of sampling sites

**Figure 2:**
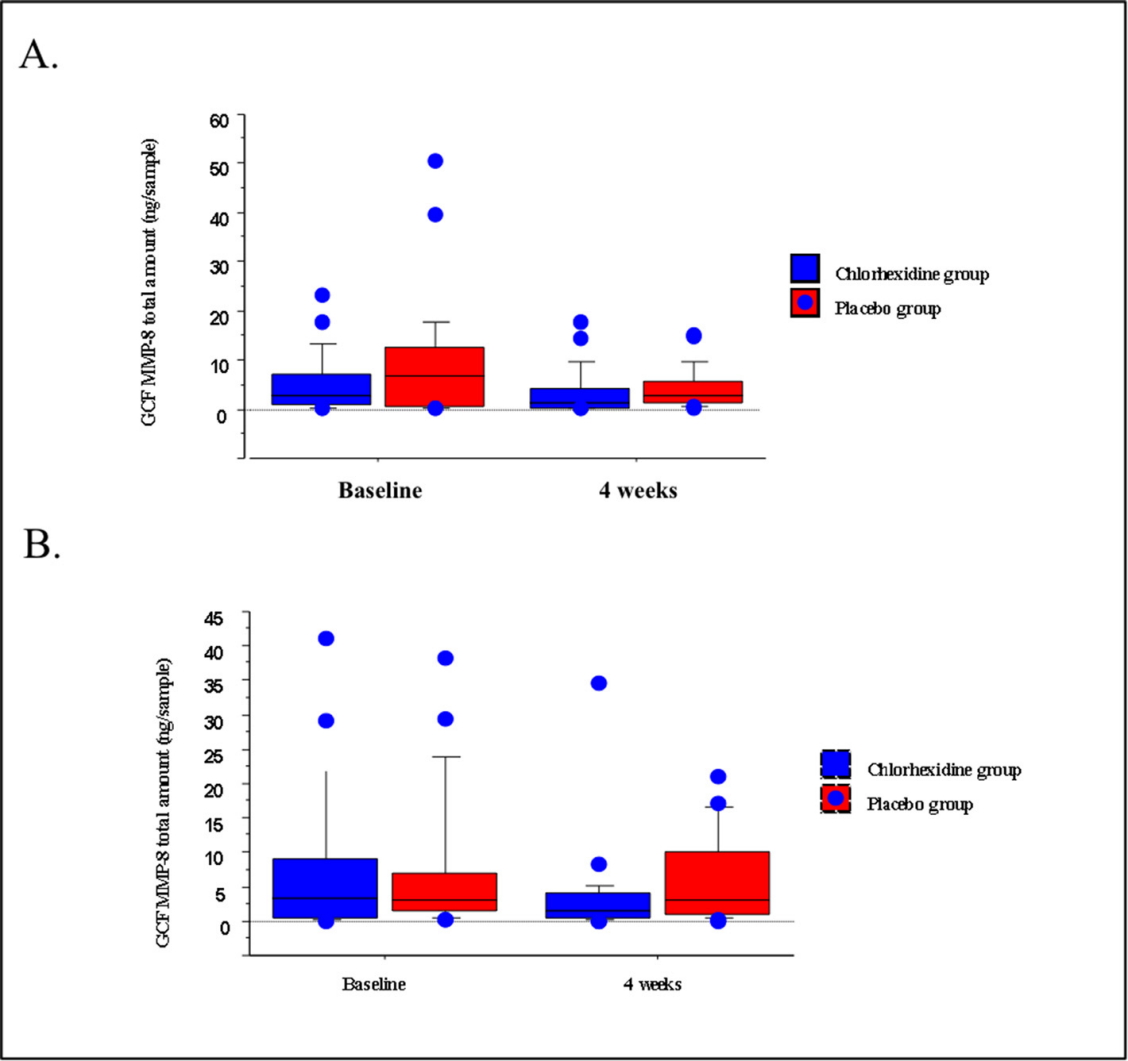
**Distributions of the total amount of GCF MMP-8 in anterior (A) and posterior (B) sampling sites in the study groups.** Box plots show medians, 25th and 75th percentiles as boxes, 10th and 90th percentiles as whiskers. Outside values are shown as solid circles.

**Figure 3:**
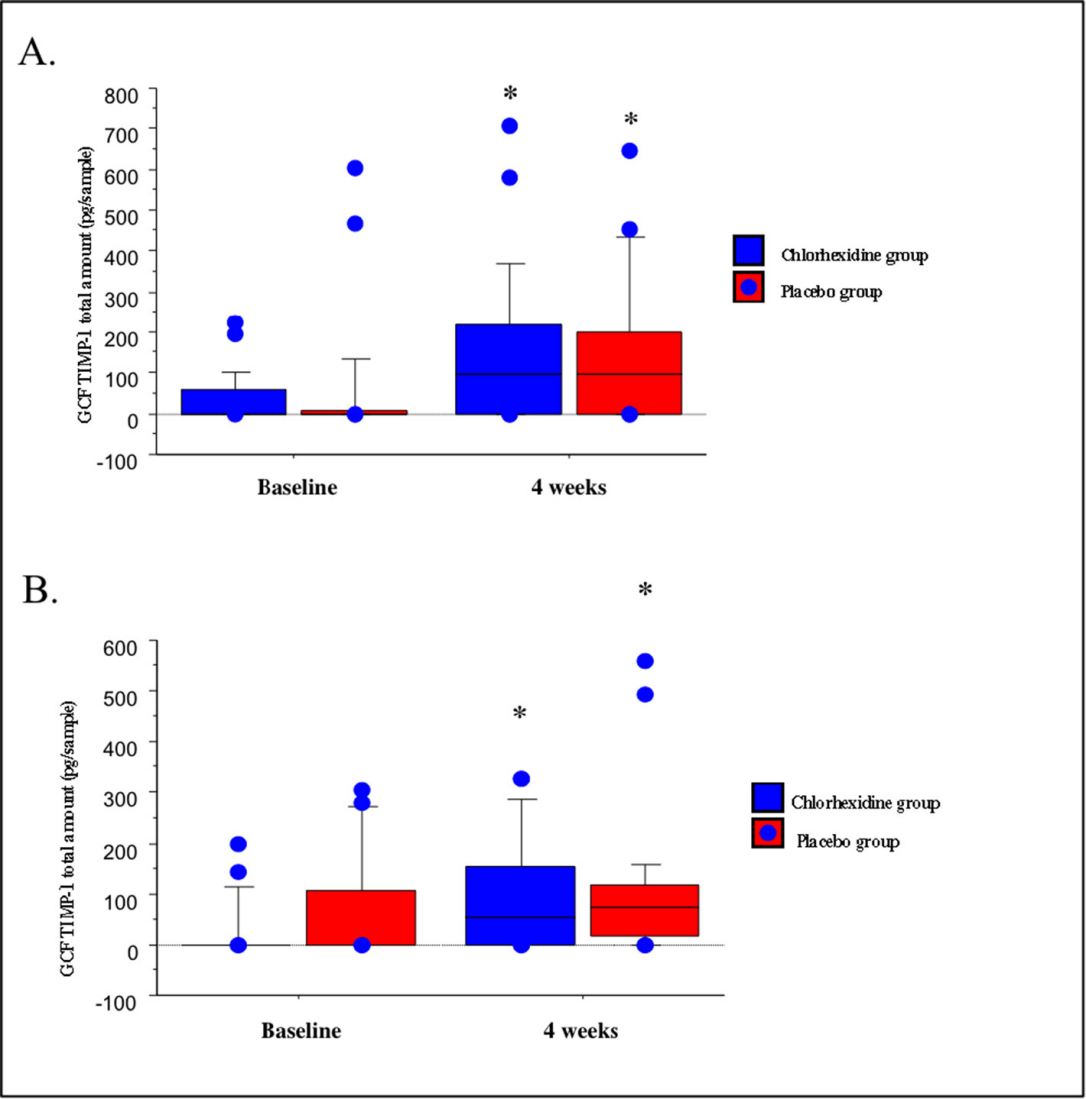
**Distributions of the total amount of GCF -TIMP-1 in anterior (A) and posterior (B) sampling sites in the study groups.** *significantly different from baseline (p < 0.05). Box plots show medians, 25th and 75th percentiles as boxes, 10th and 90th percentiles as whiskers. Outside values are shown as solid circles.

Figure 2A and B show GCF MMP-8 total amounts of anterior and posterior sites in both study groups at baseline and four weeks. At baseline, there were no significant differences in GCF MMP-8 total amounts of both anterior and posterior sampling sites between CHX and placebo groups (p > 0.05). In both CHX and placebo groups, GCF MMP-8 total amounts of anterior and posterior sites at four weeks were not significantly different from baseline levels (p *>* 0.05). There were no significant differences in GCF MMP-8 total amounts between the study groups at four weeks (p *>* 0.05). GCF TIMP-1 total amounts of anterior and posterior sites in both study groups at baseline and four weeks are shown in Figure 3. There were no significant differences in GCF TIMP-1 total amounts of both anterior and posterior sampling sites between CHX and placebo groups at baseline (p > 0.05). GCF TIMP-1 total amounts of anterior and posterior sites at four weeks were higher compared to baseline in both CHX and placebo groups (p < 0.05). There was no significant difference in GCF TIMP-1 total amount between the study groups at four weeks (p *>* 0.05) (Figure 3). When the data was calculated as concentration, similar pattern was obtained for GCF MMP-8 and TIMP-1 levels in both intergroup and intragroup comparisons (data not shown).

### Adverse effects and treatment compliance

Adverse event information such as pain, sensitivity, change in the taste perception, mucosal ulcerations, discolorations were recorded. Of the 25 subjects who rinsed their mouth with CHX mouthrinse, 5 of them experienced taste disturbances and 14 patient showed discolouration of teeth and/or tongue. Three subjects had mucosal ulcerations in CHX group.

## Discussion

In this short term, randomized, placebo controlled clinical study, which is a continuation of previously published studies [31,34], the effectiveness of CHX as an adjunct to daily plaque control on both clinical and GCF MMP-8 and TIMP-1 levels were evaluated in gingivitis patients. On the basis of the present findings, it can be concluded that adjunctive CHX provides no additional benefit over daily plaque control on GCF MMP-8 levels and periodontal parameters except on plaque accumulation at posterior sites in gingivitis patients. GCF TIMP-1 levels were increased similarly in CHX and placebo groups at 4 weeks when compared to baseline. In other words, daily plaque control procedures showed no significant effects on the GCF MMP-8 levels in plaque-associated gingivitis but increased GCF TIMP-1 levels regardless of CHX usage.

Our previous study showed that CHX mouthrinse provided an additional beneficial effect on the degree of supragingival plaque while both rinses were comparable in the reduction of gingival inflammation when used as adjunct to subjects’ oral hygiene procedures [31]. In the present study, the periodontal parameters of the sampling sites in both groups were not different at 4 weeks compared to baseline except for the PI of posterior sampling sites. Posterior sampling sites of CHX group had significantly lower PI values compared to those sites of placebo group at four weeks. In our previous study PI scores of whole mouth and sampling sites were found to be lower in CHX group than those of placebo at 4 weeks [31]. Taken together with the results of the previous study we can conclude that both rinses are comparable in the reduction of gingival inflammation when used as adjunct to oral hygiene procedures, but CHX mouthrinse have an additional beneficial effect on the degree of supragingival plaque. The results by Brownstein et al. showed that CHX mouthrinse in addition to daily plaque control without initial prophylaxis reduced plaque significantly, but gingival index scores remained unchanged after 2 months in patients with moderate to severe gingivitis [46]. However, Corbet observed no significant differences in plaque index scores in patients with gingivitis while significant difference was found between CHX and control groups in gingival bleeding scores after 3 months [5]. In other study Zanatta et al. reported little antiplaque and antigingivitis effect of CHX mouthrinse on previously plaque covered surfaces and pointed out to the importance of removing established biofilm before CHX usage [47]. Lower PI scores at the posterior sites in CHX group in the present study could reflect the adjunctive benefit of CHX at sites where mechanical plaque control is more difficult than the anterior sites. On the other hand, the present study failed to show significant differences between test and control mouthrinses in the reduction of gingival inflammation after four weeks. In other words, CHX, despite/although exerting ability to inhibit MMPs and their oxidative activation as well as to prevent oxidative inactivation of TIMP-1 and also α1-antitrypsin [9,36-38] was not effective in decreasing gingival inflammation to a statistically significantly greater extent than the placebo, which might be due to the short observation period of our study.

Pathogens in microbial dental plaque are capable of stimulating host cells to recruit into periodontal tissues [9]. As inflammatory cells such as polymorphonuclear neutrophils, which play an important role in the development of inflammatory injury, infiltrate the periodontal tissues. They begin to release their MMPs, which are considered among the indirect mechanisms of tissue destruction [9]. Hernandez et al. showed that GCF MMP-8 levels were related to progression episodes and treatment responses in patients with chronic periodontitis [19]. Researchers measured the GCF MMP-8 levels in chronic periodontitis patients both active and inactive sites before and after periodontal treatment, and they found decreased GCF MMP-8 levels after periodontal treatment except the active sites [19]. It was demonstrated that CHX chip application following SRP was beneficial in improving periodontal parameters and reducing GCF MMP-8 levels for 6 months' duration [48]. To the best of our knowledge, this is the first study evaluating the effect of the CHX mouthrinse on resolution of gingival inflammation in gingivitis patients by both clinical periodontal parameters and GCF MMP-8 and TIMP-1 levels. In the present study, GCF MMP-8 levels were not different at 4 weeks than those of baseline in the study groups. Patients enrolled into the present study were given oral hygiene instructions except interdental cleaning, and they did not receive scaling for their teeth. Therefore all patients in the present study had interdental plaque to some extent even after 4 weeks. Furthermore supra and/or subgingival calculus in addition to interdental plaque was also found in some of the sampling sites. No reduction in GCF MMP-8 levels at 4 weeks in gingivitis patients might be explained by the fact that the presence of interdental plaque on interproximal surfaces and supra and/or subgingival calculus prevent to complete resolution of inflammation.

MMP activity can be regulated by several mechanisms such as proteolytic degradation and inactivation, by non-specific endogenous inhibitors, and especially by TIMPs [9]. In the present study, GCF TIMP-1 levels of anterior and posterior sites at four weeks were higher compared to baseline in both CHX and placebo groups. However no significant difference was found in GCF TIMP-1 levels of anterior and posterior sites at 4 weeks between the study groups. It might be stated that daily plaque control resulted in the increase of TIMP-1 levels in GCF, but CHX mouthrinse could not cause an additional increase in TIMP-1 at 4 weeks. It is well known that the CHX digluconate is the most effective antimicrobial agent against to bacteria, fungi and viruses in oral cavity. Its antiplaque effect contributes to the supragingival plaque control and antigingivitis feature had an additional beneficial effect on both the degree of inflammation and the microbial accumulation within the oral cavity [32-34,36-38]. However, it is also accepted that mouthrinses have no place in the treatment of established gingivitis or periodontitis and should only be used after supra and subgingival scaling has been carried out [47]. In the present study, the participants did not use interdental cleaning devices and did not receive scaling before the completion of the trial, and it might be stated that CHX mouthrinse could not produce an additional effects on GCF MMP-8 and TIMP levels because of the presence of interdental plaque.

We included both male and female patients in the present study. Because of females have hormonal changes, including the female patients might be consider as a limitation of the study. A drop-out rate of this clinical trial was very high. We think that this high rate of drop-outs is caused by relatively long period of mouthrinse usage, which makes the patients’ compliance difficult. Besides antibiotic usage is also very common in Turkey, and this is another reason for high drop-out rate. Additionally, the changes of the GCF MMP-8 and TIMP-1 levels were evaluated at 4 weeks. However, the studies analyzing the changes of these parameters in multiple time points during long term would be useful for better understanding the adjunctive effects of CHX mouthrinse on clinical and serological parameters.

## Conclusion

CHX mouthrinse as adjuncts to daily plaque control could be effective in reduction of plaque accumulation especially in posterior sites where mechanical plaque control is more difficult than the anterior sites. CHX usage had no significant effects on the GCF MMP-8 and TIMP-1 levels in plaque-associated gingivitis. However, daily plaque control procedures could be useful in the increase of TIMP-1 levels in GCF regardless of the use of CHX.

## Abbreviations

CHX: Chlorhexidine; MMP: Matrix metalloproteinase; GCF: Gingival crevicular fluid; TIMP: Tissue inhibitors of matrix metalloproteinase; PI: Plaque index; PBI: Papillary bleeding index; CI: Calculus index; PPD: Probing pocket depth; IFMA: Immunofluorometric assay; ELISA: Enzyme linked immunosorbent assay.

## Competing interest

There is no conflict of interest in the present study.

## Authors’ contributions

OT and SB designed the study, collected the samples, commented on the data and wrote the manuscript. TT carried out the ELISA tests and commented on the data. TS, GA and GE came up with hypothesis, commented on the data and helped to write the manuscript. All authors read and approved the final manuscript.

## Pre-publication history

The pre-publication history for this paper can be accessed here:

http://www.biomedcentral.com/1472-6831/14/55/prepub
